# Improving High-Throughput Phenotyping Using Fusion of Close-Range Hyperspectral Camera and Low-Cost Depth Sensor

**DOI:** 10.3390/s18082711

**Published:** 2018-08-17

**Authors:** Peikui Huang, Xiwen Luo, Jian Jin, Liangju Wang, Libo Zhang, Jie Liu, Zhigang Zhang

**Affiliations:** 1 Key Laboratory of Key Technology on Agricultural Machine and Equipment, Ministry of Education, South China Agricultural University, Guangzhou 510642, China; peikuihuang@stu.scau.edu.cn (P.H.); xwluo@scau.edu.cn (X.L.); 2 Department of Agricultural and Biological Engineering, Purdue University, 225 S. University St., West Lafayette, IN 47907, USA; jinjian@purdue.edu (J.J.); wang3335@purdue.edu (L.W.); zhan2693@purdue.edu (L.Z.); 3 College of Engineering, Huazhong Agricultural University, Wuhan 430070, China; liujie@mail.hzau.edu.cn

**Keywords:** high-throughput phenotyping, close-range hyperspectral camera, low-cost depth sensor, fusion, plant 3D model

## Abstract

Hyperspectral sensors, especially the close-range hyperspectral camera, have been widely introduced to detect biological processes of plants in the high-throughput phenotyping platform, to support the identification of biotic and abiotic stress reactions at an early stage. However, the complex geometry of plants and their interaction with the illumination, severely affects the spectral information obtained. Furthermore, plant structure, leaf area, and leaf inclination distribution are critical indexes which have been widely used in multiple plant models. Therefore, the process of combination between hyperspectral images and 3D point clouds is a promising approach to solve these problems and improve the high-throughput phenotyping technique. We proposed a novel approach fusing a low-cost depth sensor and a close-range hyperspectral camera, which extended hyperspectral camera ability with 3D information as a potential tool for high-throughput phenotyping. An exemplary new calibration and analysis method was shown in soybean leaf experiments. The results showed that a 0.99 pixel resolution for the hyperspectral camera and a 3.3 millimeter accuracy for the depth sensor, could be achieved in a controlled environment using the method proposed in this paper. We also discussed the new capabilities gained using this new method, to quantify and model the effects of plant geometry and sensor configuration. The possibility of 3D reflectance models can be used to minimize the geometry-related effects in hyperspectral images, and to significantly improve high-throughput phenotyping. Overall results of this research, indicated that the proposed method provided more accurate spatial and spectral plant information, which helped to enhance the precision of biological processes in high-throughput phenotyping.

## 1. Introduction

Accelerating the process of breeding crops and ensuring their production is a critical step to solving the global food security problem by 2050, where a world population of 9.7 billion is projected [1]. For that purpose, high-throughput phenotyping platforms have recently been developed to solve this problem [2]. Hyperspectral imaging is a technique in high-throughput phenotyping platforms, to collect data for quantitative studies of complex traits related to the crop growth, yield, adaptation to biotic or abiotic stress (disease, insects, drought and salinity), land cover classification, and the separation of varieties in remote sensing [3]. Gonzalezdugo, V et al. [4] used high-resolution hyperspectral and thermal airborne imagery, to assess physiological conditions in the context of wheat phenotyping. Thomas, S et al. [5] summarized the benefits of hyperspectral imaging for plant disease detection and plant protection, in a technical perspective. Sytar, O et al [6] mentioned the application of hyperspectral imaging techniques, for improving salt stress tolerance. In close-range setups, it is used to detect the stress processes and defense reactions of a single plant [7,8,9,10]. Since hyperspectral imaging is able to detect the deviations in plant-physiological parameters non-destructively, a large number of plants were observed to describe phenotypical characteristics resulting from the interaction of genotypes with various environmental conditions. The importance of an accelerated and automated phenotyping of plants has grown significantly in recent years [10].

Currently, the research on phenotyping focuses on analysis based on the measurements of hyperspectral information, which can be used to compute various indexes (e.g., Normalized Difference Vegetation Index), to indicate plant biochemical contents (chlorophyll, anthocyanin, et al.). However, the complex geometry of plants (leaf angle, curvature, and self-shading) and their interaction with the illumination setting, severely affects the spectral information obtained [11]. Furthermore, plant structure (such as stalk width), leaf area, and leaf inclination distribution are critical indexes that have been widely used in multiple plant models, for example, the PROSPECT + SAIL model [12,13,14,15]. Nowadays, the processing of hyperspectral images including top view and side view, usually averages one whole plant into one value, due to the lack of 3D information. The combination of hyperspectral images and 3D point clouds, is a promising approach to solve these problems. Therefore, methods that focus on the fusion of hyperspectral images and 3D point clouds are very important for high-throughput phenotyping.

### 1.1. Previous Work

#### Close-Range Hyperspectral Camera

Hyperspectral sensors are often designed as line scanning cameras that break down the spectral composition of a 1D pixel line onto a 2D CCD (Charge-coupled Device) array, which records one spatial dimension and one spectral dimension. Consequently, a 2D hyperspectral image is composed of consecutively recorded lines [16]. Currently, the pushbroom sensor design provides the highest spectral and spatial resolutions and is the most widely used hyperspectral camera [11]. However, the well-established calibration methods for pinhole cameras are not applicable to pushbroom cameras because every line of the recorded image is the result of a unique exterior orientation, including projection center and rotation matrix. Therefore, a specific approach with a suitable setup is needed for the hyperspectral camera.

Most publications based on the line scanning camera model, focus on the scale of observations from air or spacecraft [17,18]. However, close-range applications offer higher stability of the sensor trajectory and the availability of additional orientation information, which makes them more widely used in phenotyping. Some researchers focused on the wavelength dependent spatial distortion of the current close-range hyperspectral pushbroom cameras. Špiclin et al. [19] (pp. 2813–2818), represented the distortion via a wavelength-dependent cubic spline, with a projective transformation between the image plane and the sensor plane. Lawrence et al. [20] (p. 513), calibrated the camera radio-metrically and removed smiling and keystone effects via the Geometric Control Point method, using polynomials. However, their approaches did not describe the geometric imaging characteristics of a hyperspectral camera; and therefore, did not support the combination with 3D information. Under the assumption of an acceleration-free linear movement without rotations, Gupta and Hartley [21] (pp. 963–975) proposed a simple and compact method to calibrate pushbroom cameras, which comprised the interior and exterior parameters of pushbroom cameras and was like the perspective projection matrix. Behmann et al. [16] (pp. 172–182) proposed a pushbroom camera model, based on a designed reference gauge. They considered the rotations along the movement direction, and non-linear movements occurred during the imaging, which made it suitable for application on close-range plant phenotyping. However, the calculations of the calibration models mentioned above are complex and time-consuming. Moreover, as to the combination of spectral and spatial information in close-range hyperspectral imaging, only two of the parameters in the perspective projection matrix are needed.

In this paper, we added the examination of the reference gauge and proposed a novel and simple geometric calibration method, for a close-range hyperspectral pushbroom camera using the reference objects to combine spectral and spatial information.

### 1.2. Combination of Hyperspectral Images and 3D Point Clouds

Capturing the 3D geometry parameters of plants is a common technique in plant science, which can be applied for the measurements of canopy structure, height, leaf angle distributions, root architecture, and shoot structure across various scales, ranging from laboratory to greenhouse and field [22,23,24]. Different kinds of sensor techniques, such as the stereo camera system [25], terrestrial lidar [26], and structured light approaches or laser triangulation for close-up scanning, can be used to acquire 3D geometry [27,28]. Extremely high precision sensor systems, with high resolution on the scale of plots [24] and single plant [28] are available. Even the low-cost depth sensors, such as Kinect V2 (Xbox, Microsoft, Redmond, WA 98052, USA), reach usable levels of accuracy at the mm-scale. Therefore, combining the hyperspectral images and the 3D point clouds is reasonable.

Liang et al. [29] (pp. 172–177) observed a plant from multiple viewpoints with a full frame hyperspectral camera and used detectors for homologous points and the structure from motion principle to generate a 3D model. However, these perspective images need complex post-processing, which makes it unsuitable for high-throughput phenotyping platforms. A similar approach using an unmanned aerial vehicle (UAV) and a full frame hyperspectral camera, which captures all bands simultaneously, was applied to crop surfaces [30]. The resulting crop surface model, allowed us to extract plot-wise height information and integrate it into spectral analysis. Even the reflectance of vegetation under different viewing angles can be estimated at plot scale by a virtual goniometer, using a UAV [31]. However, the UAV-based methods are time-consuming and labor intensive.

The combination of hyperspectral images and 3D point clouds is very important because, the required high spatial resolution is hard to achieve only by a pushbroom camera. The description of this sensor type in the close range, requires suitable computer vision methods. Common fusion routines for the pushbroom camera, are not adapted to the close-range applications and to the sensing of plants. Therefore, in this paper we proposed a novel method to solve these problems and verify the fusion model, using indoor soybean leaf experiments with a controlled environment. Discussions and conclusions were presented to discuss the potential applications of this technology, and possible research as a next step.

## 2. Materials and Methods

### 2.1. Physical Equipments

#### 2.1.1. Hyperspectral Sensing System

**Figure 1:**
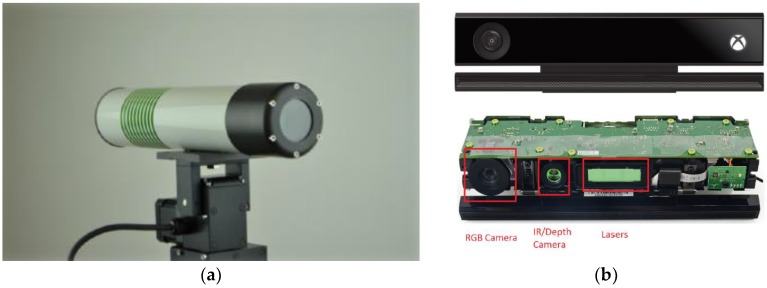
(**a**) Middleton Spectral Vision MSV 101 Hyperspectral Camera. (**b**) Kinect V2 depth sensors.

During the experiment, hyperspectral images were obtained using a Middleton Spectral Vision MSV 101 VNIR Hyperspectral Camera V10 (Middleton Research, Middleton, WI 53562, USA), as shown in Figure 1a. The sensible spectral range of this camera was 400 to 1000 nm (VISNIR), which contained 582 wavelength bands. As a pushbroom type of hyperspectral camera, the length of the spatial resolution was determined by the number of lines that were scanned during one single experiment. The width of its spatial resolution was 782 pixels per line, with a spectral resolution of 7 nm. The hyperspectral camera was mounted on a Via-Spec™ II Hyperspectral Transmission Stage, to obtain spatial images. Imaging data was recorded in a dark room to realize optimal, reproducible, and constant illumination and environmental conditions during the measurements.

#### 2.1.2. Low-Cost Depth Sensor

Kinect V2 is a second generation range sensor of Microsoft windows, shown in Figure 1b. This new version of the Kinect, is based on the time-of-flight (ToF) principle and offers a higher resolution and a wider field of view, in comparison to its predecessor. The resolution of the depth camera was 512 × 424 pixels, with an operating range between 0.5 to 4.5 m and a field of view 70.6° × 60.0°. We chose it as the depth sensor in this paper, because it is a low-cost sensor (about $100) that offers mm scale accuracy.

#### 2.1.3. Reference Gauge

**Figure 2:**
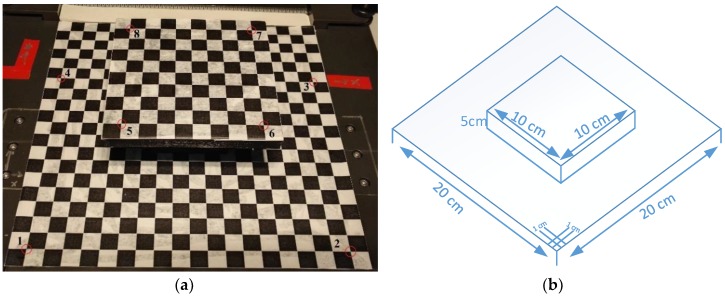
(**a**) Reference gauge with a regular chess pattern with 1 cm edge length, red circles are the selected reference points numbered from 1 to 8; (**b**) Technical drawing with size specifications.

The fusion algorithm was based on the selected reference points, with corresponding projections in the hyperspectral camera and depth sensor. For this purpose, a reference gauge was applied, which consisted of two planes, shown in Figure 2a. One large lower plane (20 cm × 20 cm), with a cuboid (10 cm × 10 cm footprint and 5 cm height) at its center with the upper plane on it. The reference points from the reference gauge, served as the bridge between 3D point clouds from Kinect V2 and image coordinates from the hyperspectral camera. Both planes were laminated with paper printed with a regular chess pattern with a 1 cm edge length, as shown in Figure 2b. Altogether, there were 260 reference points on the lower plane and 81 reference points on the upper plane.

The reference gauge was assessed using a high precision 3D printer Form 2 (Formlabs, Inc., 35 Medford St. Suite 201, Somerville, MA 02143 USA), with an accuracy of 0.2 mm. The selected reference points (Number 1–8, Figure 2a), were determined by calculating the intersection of the measured edge lines of the checkerboard to enhance the accuracy. The extracted 3D coordinates of selected points on the checkerboard were regarded as error-free, under the superior accuracy of measurement.

#### 2.1.4. Imaging Station

To set up the experiment, a system needs to be designed and utilized to support the measurement. This system was built in the lab of the Agricultural and Biological Engineering Department at Purdue University (West Lafayette, IN, USA).

**Figure 3:**
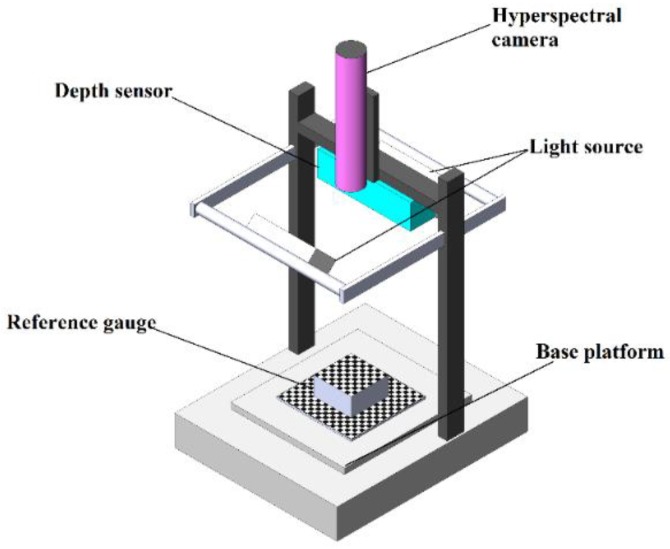
The structure of imaging station.

The system was mainly based on a high-throughput phenotyping platform, consisting of the Middleton Research Via-Spec II Hyperspectral Scanning Station, which can be programmed using Python to perform continuous imaging. The structure of the scanning station could be separated into three parts: A base platform with a designed reference gauge on it, a vertically transformable platform with halogen light source, and an imaging system on the top. As shown in Figure 3, the imaging system contained a close-range hyperspectral pushbroom camera and a depth sensor. A designed reference gauge was mounted on the base platform.

Before imaging, the 3D coordinates of the selected points on the reference gauge were measured using a Vernier caliper. Y-axis was defined as the same direction as the movement of the base platform. The X-axis was defined as the direction vertical to the Y-axis on the horizontal plane. Z-axis was defined as the vertical direction of the X-Y plane. The base platform could move using the motor which was built inside. The motor was controlled by the official Middleton software MSV.UI.Measure, which could move the whole upper platform along the Y-axis with an acceleration-free linear movement, without rotations.

After warming up the imaging system, hyperspectral images and the 3D point clouds were captured synchronously. The 3D coordinates of the selected points, the corresponding hyperspectral image coordinates, and the corresponding 3D point clouds were later used as inputs for the fusion model.

### 2.2. Plant Materials

We used soybean leaf to verify the method proposed in this paper. The soybean variety was Asgrow 30X6, and the growth stage of the plants in the experiment was V3 (V3 is a staging system we used to indicate that the plants had 3 trifoliate leaves open) with 4 nodes. The soybeans were grown in commercial potting media (Sun Gro Propagation Mix, Sun Gro Horticulture, 15831 NE 8th St, Bellevue, WA 98008, USA), in 4-inch square pots. Moreover, the soybeans were watered as needed, and fertilized weekly (Jack’s Professional 20-20-20, JR Peters Inc, 6656 Grant Way, Allentown, PA 18106, USA). The greenhouse temperature was maintained between 23 and 29 C. Supplemental light was provided by 600 W high pressure sodium bulbs, set to a 16-h photoperiod.

### 2.3. Fusion of a Close-Range Hyperspectral Camera and a Depth Sensor

The component of the fusion procedure for the close-range hyperspectral pushbroom camera and the depth sensor, was presented in this section. The reference gauge provided a high number of detectable, homogeneously distributed, and accurate reference points. Based on the selected points, the fusion model of the depth sensor and the close-range hyperspectral camera was applied.

#### 2.3.1. Semi-Automatic Coordinate Measurement 

The image coordinates of the projection for the selected points of the chess patterns in the hyperspectral image, were the input parameters of the fusion algorithm. The detectCheckerboardPoints function in Matlab 9.2, was used as a corner detector for semi-automatic point detection. Worth noting that the locations of patterns were input manually, and the semi-automated corner selection method was necessary. The points on the upper plane of the reference gauge had to be reliably separated from those on the lower plane, for a correct assignment of the 3D coordinates. Furthermore, the semi-automatic approach was more flexible if only a part of the reference gauge is observed or if the reference gauge is rotated [16].

#### 2.3.2. Alignment of Depth and Hyperspectral Sensor

Since the depth and the hyperspectral image information were captured by different kinds of sensors, the matching between hyperspectral images and 3D point clouds was required. The close-range hyperspectral pushbroom camera could be modelled as a linear camera. Like a perspective camera model, the linear camera model describes the relation between the selected points in 3D coordinates and the pixels in 2D image coordinates, as outlined in References [20,21,32,33]. To the best of our knowledge, the hyperspectral camera model often represented by a 3 × 4 projection matrix, which described the linear movement of a line scanning camera in the 3D space and the imaging characteristics of the line camera [16]. High order polynomials need to be solved in these models. However, for the combination of spectral and spatial information, just two of these parameters in the projection matrix are needed. Therefore, in this paper we proposed a novel and efficient method to fuse the depth sensor and hyperspectral camera.

**Figure 4:**
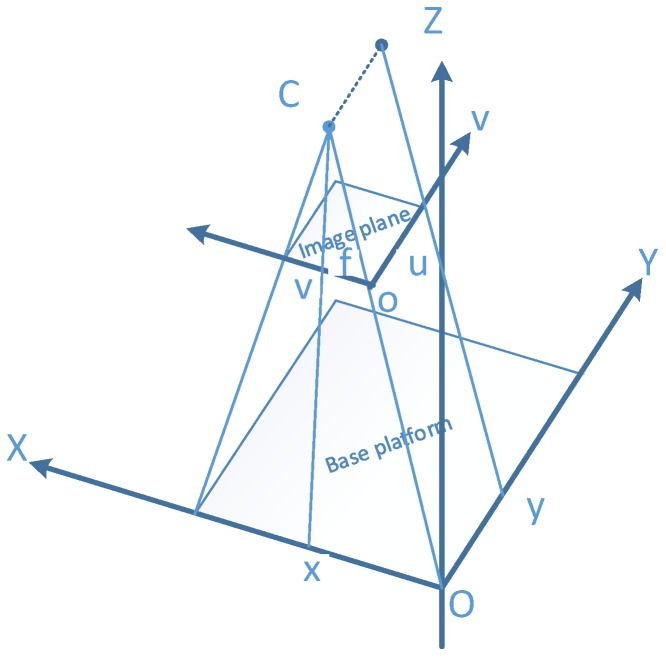
The imaging principle of the pushbroom camera model. The hyperspectral camera with focal length *f* and camera center C. The image coordinate is o-uv, while the system 3D coordinate is O-XYZ. The object point in X axis is projected to the image coordinate v, while the object point in Y axis is projected to the image coordinate u.

The resulting hyperspectral images in this paper, were created by the image coordinate u-axis (the movement direction) and the v-axis (the direction of the view plane). Worth noting that the movement of the base platform was an acceleration-free linear movement without rotation. Therefore, the pushbroom camera could be modelled by two parts, one as the view plane section, and the other one, as the movement direction section. The details of the fusion model, are shown in Figure 4.

For the view plane section, according to projection role of hyperspectral camera noted by Gupta and Hartly [21], the equation can be achieved:

(1) $$\frac{x}{D_{x}}=\frac{v}{f},$$

where x is the X axis value of object point in O-XYZ coordinate, $D_{x}$ is the depth of object point, v is the v value of object point in o-uv coordinate, and f is the focal length of the hyperspectral camera.

For the movement direction section, as the movement of the base platform was acceleration-free linear without rotation, the image coordinate u in the moving direction is the result of a parallel projection, which is only determined by scanning speed s and the moving distance y. That can be summed up as:

(2) $$u=\frac{y}{s},$$

where *u* is the v value of object point in o-uv coordinate, *y* is the Y axis value of object point in O-XYZ coordinate, and *s* is the scanning speed of the hyperspectral camera.

Therefore, to combine the spatial and spectral information between the hyperspectral camera and depth sensor, only the focal length of the hyperspectral camera and the scanning speed in pixel scale are needed.

**Figure 5:**
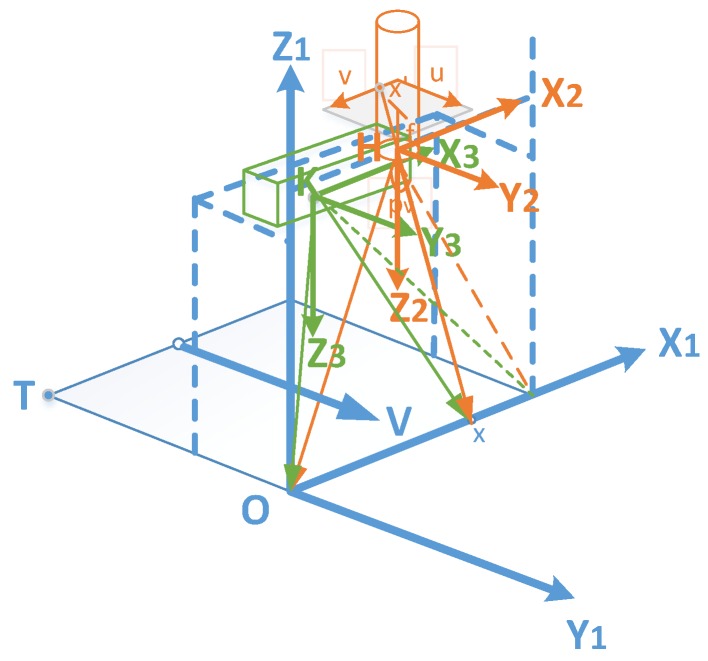
The fusion principle of pushbroom camera and depth sensor model. Starting from T, the line camera with focal length f and principal point pv is moved along V. The object point x is projected to the image point x’, with the coordinate (u; v).The coordinates of the base platform, hyperspectral camera, and Kinect V2 were O-X_1_Y_1_Z_1_, H-X_2_Y_2_Z_2_, and K-X_3_Y_3_Z_3_, respectively, which were aligned with each other.

A uniform 3D coordinate system was built to implement the fusion model. As shown in Figure 5, the uniform 3D coordinate was the base platform coordinate O-X_1_Y_1_Z_1_, which is corresponding to the system 3D coordinate O-XYZ in Figure 4. The 3D coordinate of the hyperspectral camera was based on the camera center, while, the 3D coordinate of Kinect V2 was based on the center of depth sensor, and they both aligned to system coordinate. Normally, the movement of the camera is parametrized by the starting point T, a 3D rotation matrix R, and the movement vector V with scanning speed *s*, and the movement between two pixel lines. The imaging characteristics of the line scanning camera were parametrized by the principal point (p_v_) of the sensor line (v-direction) and the focal length f. As mentioned above, the typical method needs 11 parameters in total. A selected 3D point x, is projected to the image coordinate x’.

Therefore, the fusion equation can be achieved:

(3) [ux′vx′]H=[YxsfXxZx]H,

where ($u_{x^{\prime }}$,$v_{x^{\prime }}$) were the image coordinates of the reference points, and ($X_{x}$,$Y_{x}$,$Z_{x}$) were the corresponding points in the hyperspectral camera 3D coordinate.

According to Savage, Paul G [34], the 3D transformation relationship between the hyperspectral camera and the base platform was:

(4) $${\left[\begin{array}{c} X\\ Y\\ Z \end{array}\right]}_{O}={\left[\begin{array}{c} X\\ Y\\ Z \end{array}\right]}_{H}-R_{H2O}\left[\begin{array}{c} x_{H}\\ y_{H}\\ z_{H} \end{array}\right],$$

where [$x_{H}$,$y_{H}$,$z_{H}$]^T^ was the 3D coordinate of hyperspectral camera optical center in the base platform coordinate O-X_1_Y_1_Z_1_, where the value was [153, −6.06, 755]^T^ mm in this paper. R_H2O_ is the rotation matrix from hyperspectral camera coordinate H-X_2_Y_2_Z_2_, to the base platform coordinate O-X_1_Y_1_Z_1_. It was easy to know that:

(5) $$R_{H2O}=\left[\begin{array}{ccc} 1 & 0 & 0\\ 0 & 1 & 0\\ 0 & 0 & -1 \end{array}\right],$$

Depending on the known coordinate in the O-X_1_Y_1_Z_1_ coordinate, the corresponding coordinate of reference points in the H-X_2_Y_2_Z_2_ coordinate can be calculated by Equation (5).

Same as the 3D transformation relationship between the hyperspectral camera and the base platform, the 3D transformation relationship between the Kinect V2 and the base platform was:

(6) $${\left[\begin{array}{c} X\\ Y\\ Z \end{array}\right]}_{O}={\left[\begin{array}{c} X\\ Y\\ Z \end{array}\right]}_{K}-R_{K2O}\left[\begin{array}{c} x_{K}\\ y_{K}\\ z_{K} \end{array}\right],$$

where [$x_{K}$,$y_{K}$,$z_{K}$]^T^ was the 3D coordinate of the optical center of the infrared camera in the base platform coordinate O-X_1_Y_1_Z_1_, where the value was [43, −41, 673]^T^ mm in this paper. R_K2O_ is the rotation matrix from the Kinect V2 coordinates K-X_3_Y_3_Z_3_ to the base platform coordinates O-X_1_Y_1_Z_1_, which is equal to R_H2O_.

The Equations (4) to (6) were used to calculate the 3D transformation relationship, between the hyperspectral camera and the Kinect V2 (Equation (7)):

(7) $${\left[\begin{array}{c} X\\ Y\\ Z \end{array}\right]}_{H}={\left[\begin{array}{c} X\\ Y\\ Z \end{array}\right]}_{K}+\left[\begin{array}{c} x_{H}-x_{K}\\ y_{H}-y_{K}\\ z_{K}-z_{H} \end{array}\right],$$

Up to now, the fusion model between the hyperspectral camera and the Kinect V2 can be modified as:

(8) [uv]H=[(Y+yH−yK)sf(X+xH−xK)(Z−zH+zK)]K,

Based on at least three non-planar selected points, the components of f and s can be estimated using the pushbroom camera model in Figure 4 [35]. The parameters were estimated by a least-square approach, with the assumption of uncorrelated and equal accurate coordinate measurements. To increase the fusion accuracy of the model in this paper, eight reference points were selected evenly on the upper and the lower planes of the reference gauge.

The description of pushbroom camera model contained parameters defined in the coordinate system of the selected reference points. The compact representation fusion algorithm, allowed us to apply the camera model to a similar pinhole camera model, and to support the exchange with available frameworks. Distortion, non-linear camera movement, and camera rotations in close-range applications will cause non-linearity. However, in this paper the hyperspectral camera was fixed, and the base platform was moving with an acceleration-free linear movement without rotations. Moreover, 20 images were captured one time for average analysis. Therefore, the non-linearity was ignorable in this paper.

## 3. Results & Discussion

### 3.1. Fusion Model Experiments

The experiment took place in a dark room at Purdue University. After warming up the system, hyperspectral images and 3D point clouds were captured by the computers synchronously. The setting of the Middleton software was shown in Table 1.

The hyperspectral image coordinate and system 3D coordinate of the selected reference points can be achieved, both from the hyperspectral camera and the Kinect V2 depth sensor. Worth noting that the 3D cloud points needed to be captured first because the base platform was moving during the imaging. Since the definition of image coordinate and the system 3D coordinate were both positive, to get the corresponding 3D coordinates from 3D point clouds (providing both positive and negative 3D points), a simple rectification coefficient needed to be calculated before the derivation of the fusion model. These could be abridged using the first quadrant of the Kinect V2 3D coordinate to measure the selected reference points. However, it is a special condition and needs a higher requirement for hardware platform setup. Based on at least six non-planar reference points, the components of rectification coefficients can be estimated using Cartesian coordinates principle. We used the same 8 reference points shown in Figure 2a, to calculate the rectification coefficients, and details are shown in Table 2.

For example, for X axis corrected coefficients, from reference points 1 and 2 we were able to know:

(9) $$\{\begin{array}{c} 10.87=A1\times 24.08+B1\\ -156.3=A1\times 190.87+B1 \end{array}$$

where A1 and B1, were one group of the X axis corrected coefficients. Since we had 8 reference points, four groups of corrected coefficients were available for each axis. Averages were applied to make the calculations more accurate. Finally, the corrected coefficients in this paper could be achieved:

(10) $${\left[\begin{array}{c} X\\ Y\\ Z \end{array}\right]}_{CK}={\left[\begin{array}{c} 35-X\\ 28+Y\\ 79.57+Z \end{array}\right]}_{K},$$

where CK means the corrected 3D points from Kinect V2, and the unit here was mm.

**Figure 6:**
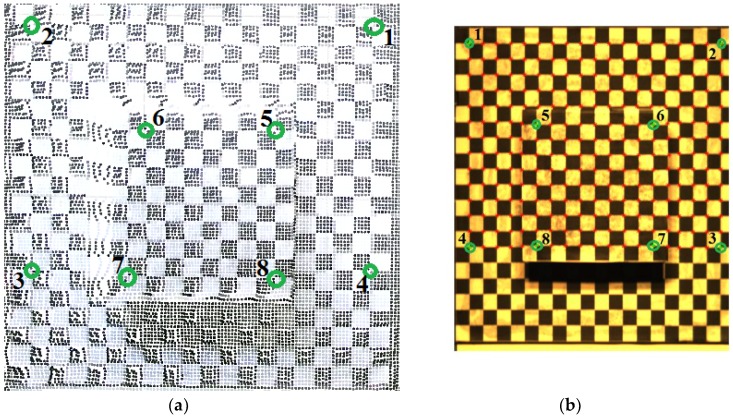
(**a**) The 3D coordinate of the selected reference points from Kinect V2 3D point clouds; (**b**) The hyperspectral image coordinate of the selected reference points achieved automatically using the corner detected method (red marks).

The fusion model between the pushbroom hyperspectral camera and the depth sensor was modified as:

(11) [uv]H=[(Y+62.94)sf(145−X)(Z+83.75)]K,

The average of the 20 images was used to calculate the fusion model. The processing hyperspectral images and 3D point clouds, were shown in Figure 6.

The corresponding image coordinates from the hyperspectral camera and 3D coordinate from Kinect V2 of the selected reference points, were shown in Table 3. The average error in the base platform reference point was 0.31 pixel in u direction and 0.94 in v direction, whilst the average error in the upper gauge was 0.18 pixel in u direction and 0.75 pixel in v direction. The accuracy of the reference points 1–4 on the base platform was 0.99 pixel, whilst the accuracy of the reference points 5-8 on the upper gauge was 0.77 pixel. The accuracy of the fusion model was in accordance with the resolution of Kinect V2 in different depth distance. Using the Equations (1) to (3), focal length f and scanning speed s were calculated, the averaged results were 2980 and 0.234 per pixel, respectively.

### 3.2. Validation Experiments

In order to verify the validity and accuracy of the fusion model proposed in this paper, soybean leaf was used to implement the validation experiment. Theoretically, the validation of the fusion model from both depth sensor information to hyperspectral camera information, and hyperspectral camera information to depth sensor information is needed. However, according to the principle of machine vision, image coordinates can be only derivated to 2D coordinate information without depth information. Therefore, we used depth information from Kinect V2 to derivate the pixel information from the close-range hyperspectral camera, to verify the fusion model proposed in this paper.

**Figure 7:**
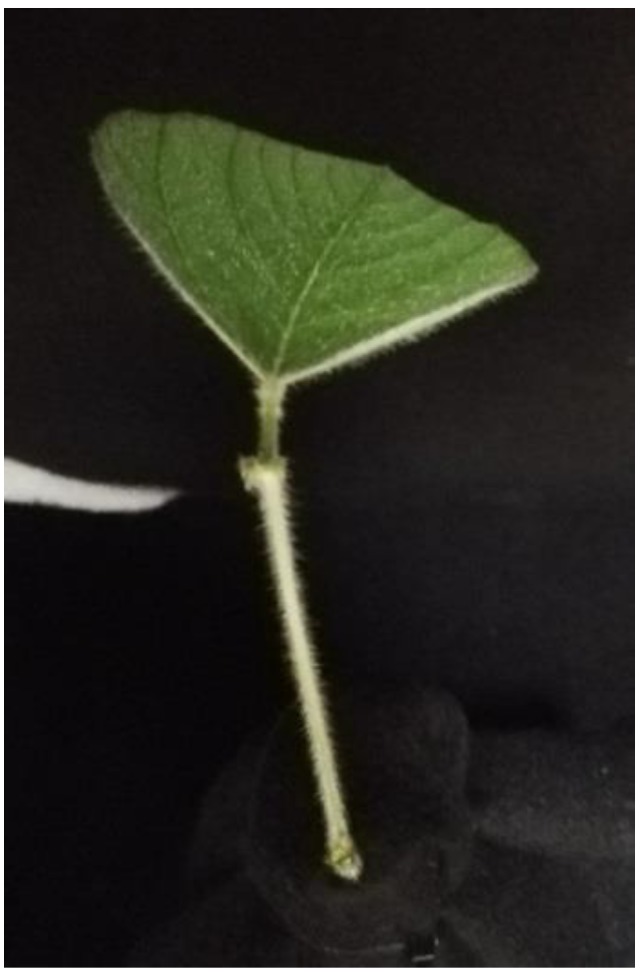
The sample soybean leaf attached to the base platform covered by a sheet of black cloth.

**Figure 8:**
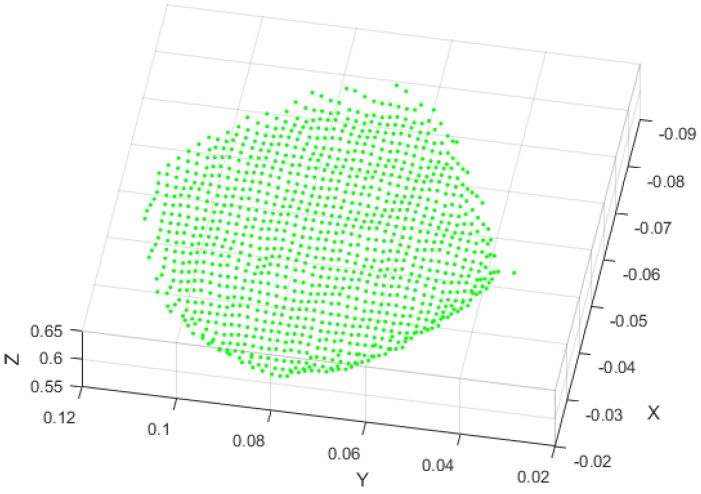
The segmentation 3-D point cloud of soybean leaf from Kinect V2.

The experiment setup was the same as in Section 3.1, but it used a piece of soybean leaf with the stem covered by a sheet of black cloth to replace the designed reference gauge. Details are shown in Figure 7. Worth noting that one complete soybean leaf contained three trifoliolate leaves and a petiole, and we only used the middle trifoliolate leaf to verify the fusion model in this paper. After Kinect V2 depth information was captured, pcshow function in Matlab 9.2 was used to plot 3-D point clouds. The 3D coordinates of soybean leaf threshold values were measured manually. The segmentation of the soybean leaf was based on these position thresholds. The threshold values in this experiment were −90 < X < 20, 35 < Y < 121.9, and 590 < Z < 630 (mm). Details are shown in Figure 8. The resolution of the Kinect V2 depth sensor was less than 3 mm within a 1 m distance, noted by Sarbolandi et.al [36]. Using the 3D distance calculation equation:

(12) $$d=\sqrt{{\left(x_{1}-x_{0}\right)}^{2}+{\left(y_{1}-y_{0}\right)}^{2}+{\left(z_{1}-z_{0}\right)}^{2}},$$

the resolution of Kinect V2 depth sensor in this experiment could be figured out as 1.54 mm.

**Figure 9:**
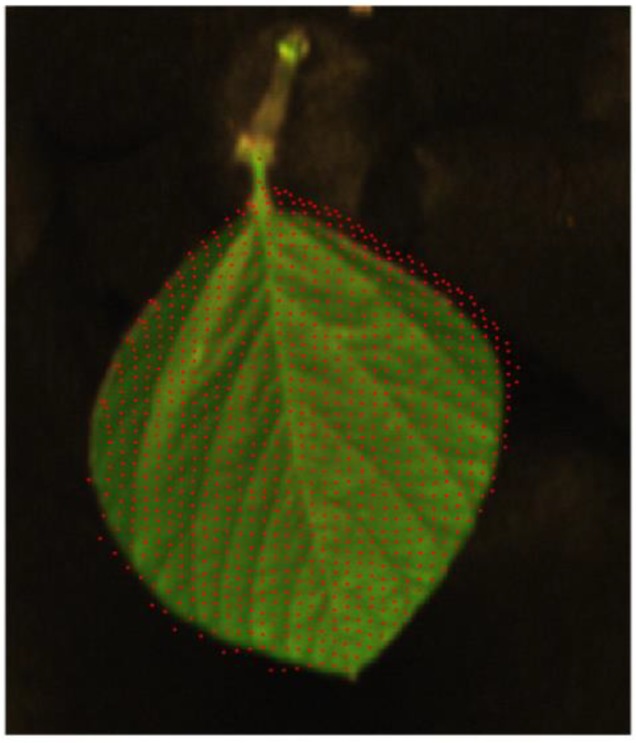
Using the fusion model proposed in this paper to derivate Kinect V2 depth information to hyperspectral image coordinate information.

After segmenting 3D point clouds of soybean leaf into the fusion model as described in Reference [13], corresponding image coordinates in the hyperspectral image could be achieved. The derivation of pixels from the Kinect V2 depth sensor was drawn as red dots on the original hyperspectral image. Details are shown in Figure 9. Mean error of depth information from Kinect V2 was 2.2 mm, and the max error was 3.3 mm in this experiment. This meant that mm scale accuracy could be achieved using the fusion model presented in this paper, with the low-cost depth sensor Kinect V2. From Figure 9, it was easy to find that the fusion model proposed in this paper was correct and effective.

### 3.3. Result Summary

The imaging system had the ability to reproduce the position of a start point of the system, which was a very important feature as it enabled the direct reuse of the fusion model for multiple images. The proposed method relied on the assumption that all bands of a surface point were projected to the same point in the image [37]. We revised this assumption in the close-range hyperspectral camera used for plant phenotyping. Details of the derivation of the fusion model based on a designed reference gauge, and the verification of the fusion model based on soybean leaf, have been presented in this paper. The movement of the hyperspectral camera was modelled as a simple linear camera model. We achieved satisfying results with pixel scale image coordinate accuracy in fusion model derivation, and 3.3 mm resolution with the low-cost Kinect V2, in close-range soybean indoor experiments with a controlled environment.

Several factors may influence the accuracy of the fusion model, including the sensor resolution, the setup of experiment platform, and the precision of data processing algorithms. Higher resolution hyperspectral cameras can achieve higher quality hyperspectral images, especially in u-axis image coordinate. With a higher resolution depth sensor, for example, 0.1 mm scale accuracy, pixel level hyperspectral image can be matched with corresponding 3D information using the fusion model in this paper. We attached the plants and gauge in the middle of the assigned start point and end point, to guarantee the uniform scanning speed when imaging the objects. Furthermore, we used an average method (captured 20 images and selected 8 reference points evenly) to minimize the error that may be caused by the corner detected algorithm.

### 3.4. Prospects of Fusion Model for Plant High-Throughput Phenotyping

**Figure 10:**
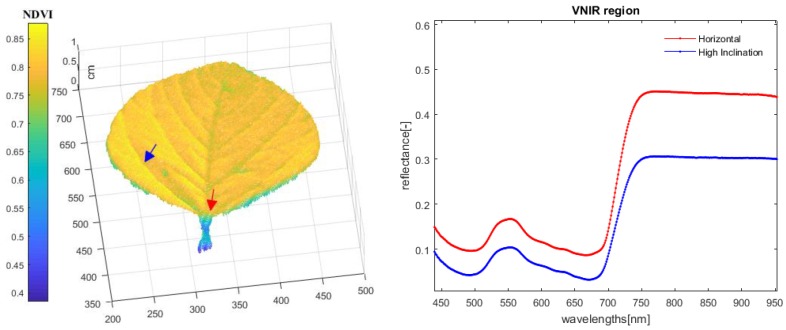
Soybean leaf hyperspectral 3D model, based on the fusion model proposed in this paper.

In this section, we discuss approaches that can add the value of the fusion model to be demonstrated in further plant observations, which are based on the linkage between hyperspectral images and 3D point clouds (depth information). This combination can be used to combine the data of more than one hyperspectral camera with depth sensors, which will help to solve the overlapping problems that were not considered in this paper. For example, a normal high-throughput greenhouse conveyor phenotyping platform usually contains two hyperspectral cameras, one is for top view imaging and one is for side view imaging, which helps to overcome overlapping during imaging. Depth sensors can attach to each hyperspectral camera, using the method proposed in this paper and build new high-throughput phenotyping platforms, which can avoid the overlapping impact and jointly analyze plants spatial and spectral features. Leaf structure, leaf area, and leaf inclination distribution can be calculated with a plant’s 3D information, which provides the possibility to build a pixel level hyperspectral 3D plant model [11,12,13,14]. The hyperspectral 3D plant model can be defined as, meshed point clouds with a hyperspectral texture. As shown in Figure 10, these models contribute a deeper understanding of the light-surface interactions at imaging plants. Moreover, it helps to enhance the research of light propagation during measurement and build a 3D reflectance model, which can be used to minimize the geometry-related effects (such as reflection of objects) in hyperspectral images. As the example shows in this paper, a 3D model of the observed plant was measured using the proposed fusion model. The 3D point clouds of the plant were transformed to the coordinate system of the hyperspectral camera model, in the first step. Then, each light beam between the moving base platform and each 3D point was examined, by intersection with the fusion model. Finally, the observed 3D points were transformed into the image coordinate and the corresponding pixel value was assigned to each point. Plants geometry parameters, such as leaf area and inclination distribution, can be calculated using the fusion model, which in the soybean leaf experiments were 32.2 cm^2^ and 63.1° inclination, respectively. With the built of soybean leaf hyperspectral 3D model, more precise (pixel level) hyperspectral analysis can be achieved. From the figure, we could easily find out that leaf angle has a severe influence on the biological processes of soybean leaf. The 3D information of plants is required when using the hyperspectral camera in a high-throughput phenotyping platform.

More and more researchers have realized the importance of plants 3D information in hyperspectral imaging. Paulus et al. [38] pointed out that the additional spatial information could be used to improve the classification accuracy of organ identification by Surface Feature Histograms, for the monitoring of plant growth. These approaches allow us to interpret the spectral information in the spatial context and help to detect plant diseases at an early stage. For example, symptoms of plant diseases may appear differently on veins and on interveinal leaf tissues. Chéné et al. [39] demonstrated that the hyperspectral 3D model can facilitate the inclusion of further imaging sensors, like thermal or fluorescence imaging. Such additional images can be projected to the same 3D model using their respective camera models and fused with the texture information at the same surface point subsequently. Vos et al. [40] mentioned that the building of the hyperspectral 3D plant model may be an important database for functional structural plant models, which links internal plant processes to the structure and the external appearance of plants. These models can be used to link experimental observations of plant phenotypes to genotypes [41,42].

## 4. Conclusions

The proposed method for combination of a close-range hyperspectral pushbroom camera and a low-cost depth sensor, is based on the pushbroom camera model and the selected reference points. The presented pushbroom camera was modelled using the view plane section and the movement direction section. Eight reference points on both the upper and lower planes were evenly selected, which were used to combine spectral information from the hyperspectral camera and spatial information from depth sensors. The validation soybean leaf indoor experiments, showed that the fusion model between the hyperspectral image system and Kinect V2 was able to fulfill the specified demands for close-range phenotyping application. The results showed that in a controlled environment, 0.99 pixel level accuracy for the hyperspectral camera and 3.3 mm accuracy for the depth sensor could be achieved, using the method proposed in this paper. We also discussed the new capabilities gained using this new method, which can allow the effects of plant geometry and sensor configuration to be quantified and modelled. The possibility of 3D reflectance models can be used to minimize the geometry-related effects in hyperspectral images; and therefore, has the potential to improve high-throughput phenotyping significantly. This method contributes to the potential of combining images from two or more (hyperspectral) sensors, and the derivation of hyperspectral 3D models.

High throughput phenotyping platforms that integrate depth sensors, hyperspectral cameras, and other cameras (such as thermography images), may fuse the recorded data automatically based on the proposed fusion model in this paper. The 3D plant models may be built by the automated sensing systems, under strictly controlled environmental conditions with a high level of reproducibility. With the 3D plant model library, a 3D white reference system may be built to improve the simulation of illumination significantly. Large amounts of 3D plant model data sets will provide more accurate analysis methods, for a high number of phenotypic traits.

## Figures and Tables

**Table 1:** The setting of the system in Middleton software.

Parameter Setting	Value
Start position (inch)	0.8
End position (inch)	9.2
Scan Speed (inch/ms)	0.55
Framerate (Hz)	60.000
Integration time (ms)	7–500

Note: the scan speed here was different from the scanning speed in fusion model, it was in distance scale.

**Table 2:** The rectification coefficients based on the reference points.

Reference Points	Kinect V2 3D Coordinate			System 3D Coordinate		
	X Axis	Y Axis	Z Axis	X Axis	Y Axis	Z Axis
1	24.08	−16.99	683	10.87	11.11	762
2	−156.3	−17.685	676.5	190.87	11.51	755.5
3	−159.55	117.55	674	191.17	141.48	753
4	23.825	116.15	677.25	11.17	141.11	756.25
5	−27.785	32.605	631.5	59.04	62.94	710.5
6	−108.1	32.3675	628.25	142.76	63.03	707.25
7	−107.65	112.4	626.75	142.79	140.26	705.25
8	−27.4878	112.5	626.25	59.11	140.26	705.25

**Table 3:** The corresponding image coordinates and 3D coordinates of the selected reference points.

Reference Points	u	v	x	y	z
1	42.24763	49.61491	24.08	−16.99	683
2	760.5121	51.39052	−156.3	−17.685	676.5
3	759.041	631.5677	−159.55	117.55	674
4	44.43668	630.1391	23.825	116.15	677.25
5	234.8244	280.9727	−27.785	32.605	631.5
6	567.7642	281.352	−108.1	32.3675	685.25
7	567.8614	626.1389	−107.65	112.4	626.75
8	235.0701	626.13	−27.4875	112.5	626.25

## References

[1] Bai G., Ge Y., Hussain W., Baenziger P.S., Graef G. (2016). A multi-sensor system for high throughput field phenotyping in soybean and wheat breeding. Comput. Electron. Agric..

[2] Li L., Zhang Q., Huang D. (2014). A review of imaging techniques for plant phenotyping. Sensors.

[3] Thenkabail P.S., Lyon J.G., Huete A. (2012). Advances in Hyperspectral Remote Sensing of Vegetation and Agricultural Croplands. Hyperspectral Remote Sensing of Vegetation.

[4] Gonzalezdugo V., Hernandez P., Solis I., Zarcotejada P. (2015). Using high-resolution hyperspectral and thermal airborne imagery to assess physiological condition in the context of wheat phenotyping. Remote Sens..

[5] Thomas S., Kuska M.T., Bohnenkamp D., Brugger A., Alisaac E., Wahabzada M. (2018). Benefits of hyperspectral imaging for plant disease detection and plant protection: A technical perspective. J. Plant Dis. Prot..

[6] Sytar O., Brestic M., Zivcak M., Olsovska K., Kovar M., Shao H. (2016). Applying hyperspectral imaging to explore natural plant diversity towards improving salt stress tolerance. Sci. Total Environ..

[7] De Jong S.M., Addink E.A., Hoogenboom P., Nijland W. (2012). The spectral response of Buxus sempervirens to different types of environmental stress—A laboratory experiment. ISPRS J. Photogramm. Remote Sens..

[8] Mahlein A.-K., Oerke E.-C., Steiner U., Dehne H.-W. (2012). Recent advances in sensing plant diseases for precision crop protection. Eur. J. Plant Pathol..

[9] Sun L., Simmons B.A., Singh S. (2011). Understanding tissue specific compositions of bioenergy feedstocks through hyperspectral Raman imaging. Biotechnol. Bioeng..

[10] Furbank R.T., Tester M. (2011). Phenomics–technologies to relieve the phenotyping bottleneck. Trends Plant Sci..

[11] Behmann J., Mahlein A.K., Paulus S., Dupuis J., Kuhlmann H., Oerke E.C., Plümer L. (2016). Generation and application of hyperspectral 3D plant models: methods and challenges. Mach. Vis. Appl..

[12] Féret J.B., Gitelson A.A., Noble S D., Jacquemoud S. (2017). PROSPECT-D: Towards modeling leaf optical properties through a complete lifecycle. Remote Sens. Environ..

[13] Jacquemoud S., Verhoef W., Baret F., Bacour C., Zarco-Tejada P.J., Asner G.P., Ustin S.L. (2009). PROSPECT+ SAIL models: A review of use for vegetation characterization. Remote Sens. Environ..

[14] Jay S., Bendoula R., Hadoux X., Féret J.B., Gorretta N. (2016). A physically-based model for retrieving foliar biochemistry and leaf orientation using close-range imaging spectroscopy. Remote Sens. Environ..

[15] Wilson R.T. (2013). Py6S: A Python interface to the 6S radiative transfer model. Comput. Geosci..

[16] Behmann J., Mahlein A.K., Paulus S., Kuhlmann H., Oerke E.C., Plümer L. (2015). Calibration of hyperspectral close-range pushbroom cameras for plant phenotyping. ISPRS J. Photogramm. Remote Sens..

[17] Kornus W., Lehner M., Schroeder M. (2000). Geometric in-flight calibration of the stereoscopic line-CCD scanner MOMS-2P. ISPRS J. Photogramm. Remote Sens..

[18] Poli D., Toutin T. (2012). Review of developments in geometric modelling for high resolution satellite pushbroom sensors. Photogramm. Rec..

[19] Špiclin Ž., Katrašnik J., Bürmen M., Pernuš F., Likar B. (2010). Geometric calibration of a hyperspectral imaging system. Appl. Opt..

[20] Lawrence K.C., Park B., Windham W.R., Mao C. (2003). Calibration of a pushbroom hyperspectral imaging system for agricultural inspection. Trans. ASAE.

[21] Gupta R., Hartley R.I. (1997). Linear pushbroom cameras. IEEE Trans. Pattern Anal. Mach. Intell..

[22] Wagner B., Santini S., Ingensand H., Gärtner H. (2011). A tool to model 3D coarse-root development with annual resolution. Plant Soil.

[23] Hosoi F., Nakabayashi K., Omasa K. (2011). 3-D modeling of tomato canopies using a high-resolution portable scanning lidar for extracting structural information. Sensors.

[24] Omasa K., Hosoi F., Konishi A. (2006). 3D lidar imaging for detecting and understanding plant responses and canopy structure. J. Exp. Bot..

[25] Biskup B., Scharr H., Schurr U., Rascher U.W.E. (2007). A stereo imaging system for measuring structural parameters of plant canopies. Plant Cell Environ..

[26] Tilly N., Hoffmeister D., Liang H., Cao Q., Liu Y., Lenz-Wiedemann V., Bareth G. (2012). Evaluation of terrestrial laser scanning for rice growth monitoring. Int. Arch. Photogramm. Remote Sens. Spat. Inf. Sci..

[27] Bellasio C., Olejníčková J., Tesař R., Šebela D., Nedbal L. (2012). Computer reconstruction of plant growth and chlorophyll fluorescence emission in three spatial dimensions. Sensors.

[28] Paulus S., Schumann H., Kuhlmann H., Léon J. (2014). High-precision laser scanning system for capturing 3D plant architecture and analysing growth of cereal plants. Biosyst. Eng..

[29] Liang J., Zia A., Zhou J., Sirault X. 3D plant modelling via hyperspectral imaging. Proceedings of the 2013 IEEE International Conference on Computer Vision Workshops (ICCVW).

[30] Bareth G., Aasen H., Bendig J., Gnyp M.L., Bolten A., Jung A., Soukkamäki J. (2015). Low-weight and UAV-based hyperspectral full-frame cameras for monitoring crops: Spectral comparison with portable spectroradiometer measurements. Photogramm. Fernerkun.

[31] Burkart A., Aasen H., Alonso L., Menz G., Bareth G., Rascher U. (2015). Angular dependency of hyperspectral measurements over wheat characterized by a novel UAV based goniometer. Remote Sens..

[32] Hui B., Wen G., Zhao Z., Li D. (2012). Line-scan camera calibration in close-range photogrammetry. Opt. Eng..

[33] Draréni J., Roy S., Sturm P. (2011). Plane-based calibration for linear cameras. Int. J. Comput. Vis..

[34] Savage P.G. (2013). Blazing gyros: The evolution of strapdown inertial navigation technology for aircraft. J. Guid. Control Dyn..

[35] Zhang Z. (1998). Determining the epipolar geometry and its uncertainty: A review. Int. J. Comput. Vis..

[36] Sarbolandi H., Lefloch D., Kolb A. (2015). Kinect range sensing: Structured-light versus time-of-flight kinect. Comput. Vis. Image Underst..

[37] Thibos L.N., Bradley A., Still D.L., Zhang X., Howarth P.A. (1990). Theory and measurement of ocular chromatic aberration. Vis. Res..

[38] Paulus S., Dupuis J., Mahlein A.-K., Kuhlmann H. (2013). Surface feature based classification of plant organs from 3D laserscanned point clouds for plant phenotyping. BMC Bioinf..

[39] Chéné Y., Rousseau D., Lucidarme P., Bertheloot J., Caffier V., Morel P., Belin É., Chapeau-Blondeau F. (2012). On the use of depth camera for 3D phenotyping of entire plants. Comput. Electron. Agric..

[40] Vos J., Evers J., Buck-Sorlin G., Andrieu B., Chelle M., De Visser P. (2010). Functional–structural plant modelling: A new versatile tool in crop science. J. Exp. Bot..

[41] Xu L., Henke M., Zhu J., Kurth W., Buck-Sorlin G. (2011). A functional–structural model of rice linking quantitative genetic information with morphological development and physiological processes. Ann. Bot..

[42] Kuska M., Wahabzada M., Leucker M., Dehne H.-W., Kersting K., Oerke E.-C., Steiner U., Mahlein A.-K. (2015). Hyperspectral phenotyping on the microscopic scale: Towards automated characterization of plant–pathogen interactions. Plant Methods.

